# Significance of Raffinose Family Oligosaccharides (RFOs) metabolism in plants

**DOI:** 10.1007/s44307-024-00022-y

**Published:** 2024-03-19

**Authors:** Huan Liu, Fan Wang, Baohui Liu, Fanjiang Kong, Chao Fang

**Affiliations:** School of Life Sciences, Innovative Center of Molecular Genetics and Evolution, Guangzhou University, Guangzhou, 510006 China

**Keywords:** RFOs, Galactinol synthase, Raffinose synthase, Stachyose synthase, α-Galactosidase, Stress

## Abstract

Raffinose Family Oligosaccharides (RFOs) are a kind of polysaccharide containing D-galactose, and they widely exist in higher plants. Synthesis of RFOs begins with galactinol synthase (GolS; EC 2.4.1.123) to convert myo-inositol into galactinol. The subsequent formation of raffinose and stachyose are catalyzed by raffinose synthase (RS; EC 2.4.1.82) and stachyose synthase (STS; EC 2.4.1.67) using sucrose and galactinol as substrate, respectively. The hydrolysis of RFOs is finished by α-galactosidase (α-Gal; EC 3.2.1.22) to produce sucrose and galactose. Importance of RFOs metabolism have been summarized, e.g. In RFOs translocating plants, the phloem loading and unloading of RFOs are widely reported in mediating the plant development process. Interference function of RFOs synthesis or hydrolysis enzymes caused growth defect. In addition, the metabolism of RFOs involved in the biotic or abiotic stresses was discussed in this review. Overall, this literature summarizes our current understanding of RFOs metabolism and points out knowledge gaps that need to be filled in future.

## Introduction

**Fig. 1:**
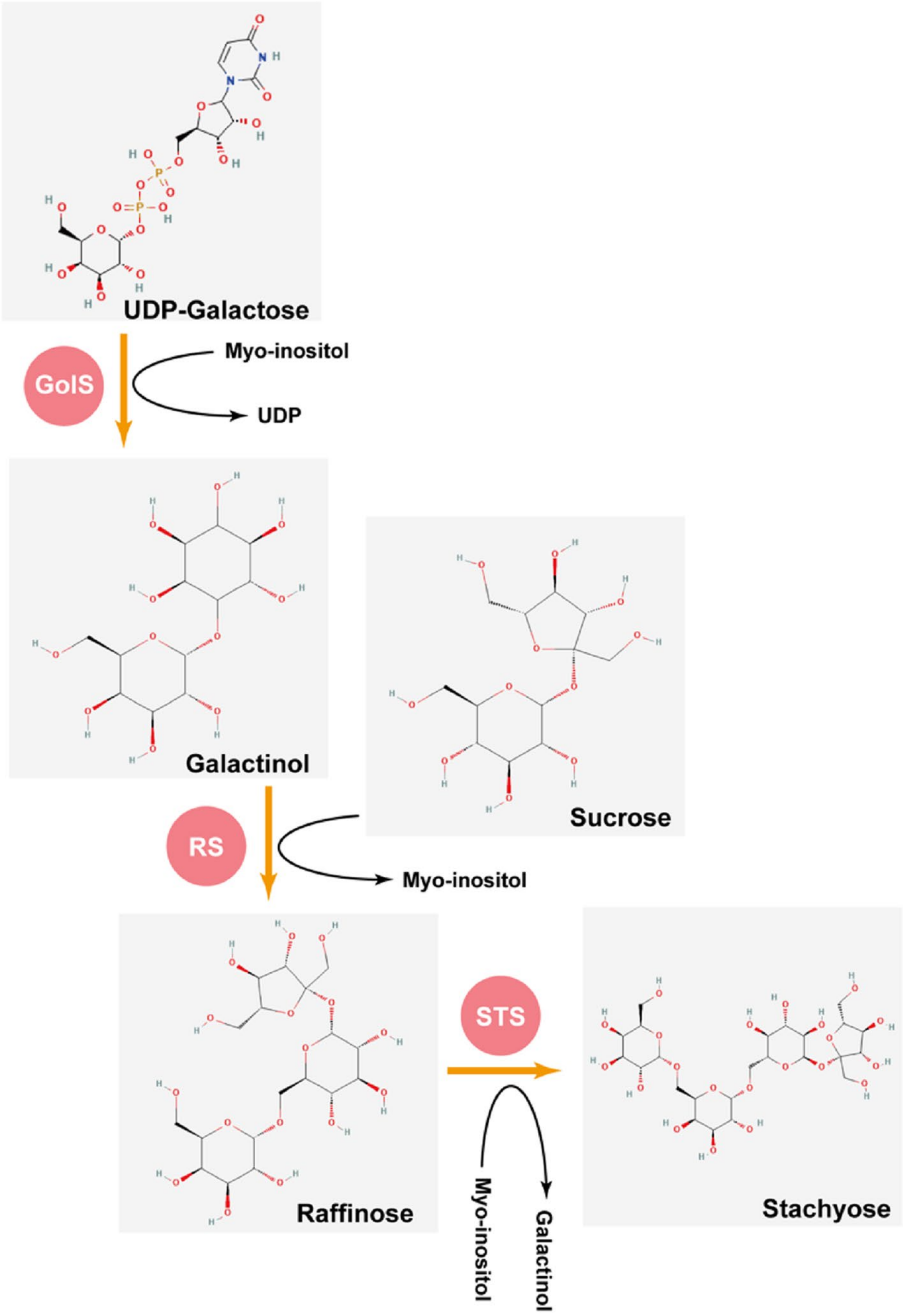
Proposed model of RFOs synthesis. The RFOs pathway is represented with key genes, namely, GolS, RS and STS. GolS: galactinol synthase; RS, raffinose synthase; STS, stachyose synthase. The structure of sugars was downloaded from NCBI (https://pubchem.ncbi.nlm.nih.gov/)

RFOs are the extension of sucrose with α-1, 6-galactosyl that happened frequently in higher plants. As non-structural and non-reducing sugars, they accumulate large quantities with unaffected primary metabolism (Peters et al. 2007). The synthesis of galactinol by the galactinol synthase (GolS) using myo-inositol and UDP-galactose marked the beginning of RFOs biosynthesis. Subsequently, raffinose, the ubiquitous existing form in all plants, is produced by raffinose synthase (RS; galactinol-sucrose galactosyltransferase) to transfer a galactosyl moiety from galactinol to sucrose to form an α-1,6-galactosidic linkage, and the stachyose is the product of stachyose synthase (STS; galactinol-raffinose galactosyltransferase) with galactinol and raffinose as substrate to transfer the galactosyl moiety from galactinol to the C6 position of the galactose unit in raffinose (Holthaus and Schmitz 1991; Peterbauer and Richter 1998; Hoch et al. 1999; Peterbauer et al. 2002; Gangl et al. 2015; Sanyal et al. 2023) (Fig. 1). In addition, the higher molecular weight RFOs also present in plants, such as verbascose and ajugose (Kotiguda et al. 2006; Dai et al. 2014), but different from the biosynthesis of raffinose and stachyose, the higher RFOs oligomer biosynthesis follows galactinol independent pathway, with galactan: galactan galactosyltransferase (GGT) to realize chain elongation by utilizing RFOs as galactosyl donors and acceptors in *Ajuga reptans* (Haab and Keller 2002; Tapernoux-Lüthi et al. 2004; Elango et al. 2022). Raffinose and stachyose are the main objects in this review. In cucurbit fruits, the monosaccharides nearly replaced RFOs, indicating the importance of an enzyme in hydrolyzing RFOs (Hubbard et al. 1989). The digestion of RFOs in plants is mediated by α-galactosidase (α-Gal) to produce sucrose and galactose (Keller and Pharr 1996).

Galactinol synthase, also named inositol-3-a-galactosyltransferase, belongs to the eukaryotic glycosyltransferase family (GTs, EC 2.4.x.y), specifically GT 8 group (Sengupta et al. 2012). GolS are relatively conserved in the monocotyledonous compared with the dicotyledonous in the plant kingdom, and the phylogenetic configuration of RS among plant kingdom was correlated to GolS, while the STS is disarrayed compared to GolS and RS (Sengupta et al. 2015). The same diversification pattern of GolS and RS probably indicates a similar evolution manner whereas STS may not have coevolved with them. Botanists also presumed that the RFOs biosynthetic scheme was split into two parts according to their evolution: the raffinose synthesis and a higher degree of polymerization RFOs synthesis (Li et al. 2017).

α-galactosidase, also named α-D-galactoside galactohydrolase or melibiose, plays an exoglycosidase role in the hydrolysis of the terminal nonreducing α-galactosyl moieties from galactic oligosaccharides, polymeric galactomannans, glycolipids or glycoproteins (Zhang et al. 2021). Eukaryotic α-galactosidases belong to the glycoside hydrolase families GH 27 (Fujimoto et al. 2003). Based on their activity dependent on pH, α-Gals were classified into acid α-Gals and alkaline α-Gals families. The sequence of the alkaline α-Gal is different from that of the acidic isoforms, but it is highly homologous to RS and STS (Carmi et al. 2003). Each α-Gal group contains proteins from monocotyledons and dicotyledons, and α-Gal of these monocotyledons or dicotyledons is always aggregated into small subgroups, indicating that the ancestors of acid and alkaline α -Gals existed prior to the separation of monocotyledons and dicotyledons.

Apart from the involvement of RFOs in signal transduction (Stevenson et al. 2000; Xue et al. 2007), membrane trafficking (Thole and Nielsen 2008), mRNA export (Okada and Ye 2009) and osmoprotectants during seed desiccation (Saravitz et al. 1987; Downie et al. 2003), the phloem loading in source, transport and storage of carbon in sink also need the participation of RFOs in plants (Kannan et al. 2021; Yan et al. 2022). In addition, RFOs would accumulate in vegetative tissues to response to biotic or abiotic stresses in many variabilities such as raffinose, stachyose, verbascose and ajugose (Jing et al. 2023). In this review, we will focus on the regulation of GolS, RS, STS and α-Gal to the RFOs metabolism, especially the phloem loading, phloem unloading and stress responses.

### RFOs phloem loading in source leaves

In higher plants, the continuous and balanced growth depends mainly on the photosynthesis in leaves and transport of nutrients in the phloem. In most plants, the final production of photosynthesis is sucrose, and it acts as both in glycolysis or carbon and energy supply for plant growth and development (Ho and Thornley 1978; Rocher et al. 1989). Sucrose phosphate synthase (SPS; EC 2.4.14) converts the fructose-6-phophate (F6P) and uridine diphosphate -glucose (UDPG) into UDP and sucrose-6-phosphate (S6P), which was catalyzed by sucrose phosphatase (SPP) to produce sucrose (Huber and Huber 1996). Sucrose subsequently enters the phloem of leaves either in an apoplastic or through the symplastic manner in most plants, which is called “phloem loading”.

**Fig. 2:**
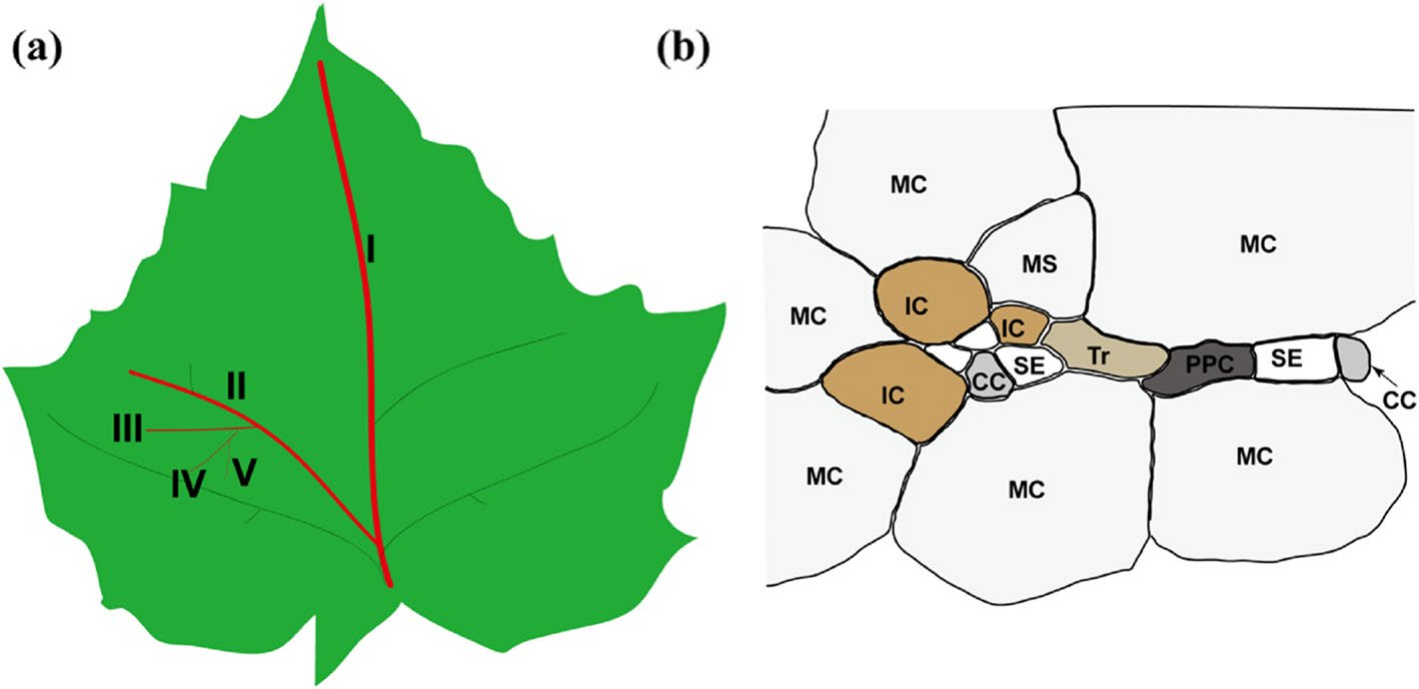
The structure of cucumber leaf veins and phloem loading. **a** Representation of cucumber vein orders in leaves. b The anatomic structure of vascular bundle in the fifth vein. MC, mesophyll cell; IC, intermediary cell; CC, companion cell; SE, seive element; Tr, tracheids cell; PPC, phloem parenchyma cell

The loading site of photoassimilate in source leaves is minor veins (Fig. 2a), which are immature and do not role in phloem unloading in sink leaves but mature after the sink to source transition (Turgeon and Webb 1976; Turgeon 1987). Three phloem loading strategies, including one passive and two active manners, existed in higher plants (Turgeon and Medville 2011). Passive phloem loading is that the sucrose moves passively into the sieve elements/companion cells (SE/CC) complex driven by high concentrations of photoassimilates in the cytosol of mesophyll cells (Turgeon and Medville 1998; Reidel et al. 2009; Rennie and Turgeon 2009), such as in poplar trees (Zhang et al. 2014a, b). One active phloem loading is via an apoplastic step against a concentration gradient, especially in herbaceous species, and it needs the participation of sugar transporters. Sucrose transporters (SUTs) uptake sucrose from apoplast against a concentration gradient to cells after the Sugar Will Eventually be Exported Transporters (SWEETs) translocate sucrose into apoplast following the concentration gradient (Chen et al. 2012; Griffiths et al. 2016). The other active phloem loading is symplastic, such as in *Cucurbitaceae*, *Lamiaceae*, *Oleaceae* and *Onagraceae*, sucrose would be further catalyzed to synthesize RFOs in unusual companion cells named intermediary cells (IC, with abundant and anatomically distinct plasmodesmata linking them to the bundle sheath cells) of leaves minor veins (Zhang and Turgeon 2018) (Fig. 2b). This phloem loading manner depends on the synthesis of RFOs in IC called polymer trapping model, and the RFOs would not diffuse in the opposite direction to back into the bundle sheath for the narrowness of specialized plasmodesmatal channels (Turgeon and Gowan 1990; Turgeon et al. 1993; Comtet et al. 2017).

The process of RFOs metabolism was widely studied in *Arabidopsis thaliana* (Iftime et al. 2011; Jang et al. 2018), *Ajuga reptans* (Peters and Keller 2009), legume seeds (Peterbauer and Richter 1998; Blöchl et al. 2005), *Coleus blumei* (Turgeon and Gowan 1990), and *Cucurbitaceae* (Ma et al. 2019; Ren et al. 2021). In RFOs translocating plants, the major sugars in phloem sap are raffinose and stachyose, especially the stachyose (Lü et al. 2017). As one of the principal metabolites of the classical RFOs biosynthesis pathway, galactinol was proved to be synthesized by GolS using myo-inositol and UDP-galactose. Immunocytochemical methods suggested that the GolS was located in minor vein ICs of mature leaves but not the ordinary companion cells (OCCs) of large veins or in ICs of young leaves in *Cucurbita pepo* (Beebe and Turgeon 1992). Similarly, the expression was limited in minor veins when expressing the melon GolS (*CmGAS*) promoter in Arabidopsis and cultivated tobacco (Haritatos et al. 2000). Downregulation of two ICs localized GolS (*VpGAS1* and *VpGAS2*) in *Verbascum phoeniceum* caused disrupted phloem loading but suppressed the expression of *VpSUT1* did not trigger any problem of phloem loading, suggesting typical polymer trapping model of *V. phoeniceum* (McCaskill and Turgeon 2007; Zhang and Turgeon 2009). Although it seems unlikely that the slightly different sizes of sugars could be distinguished within such fine selectivity of plasmodesmata between bundle sheath cells and ICs (Liesche and Schulz 2013), subsequent reports demonstrate that the convective sweeping of RFOs from mesophyll to phloem plays a positive and critical role in the segregation of RFOs to prevent them diffusion into bundle sheath cells direction (Comtet et al. 2017).

The RS and STS genes were highly expressed in watermelon leaves, suggesting the synthesis of RFOs here (Guo et al. 2015). In cucumber, the mature leaf veins were divided into 5 grades, the main veins contain most OCCs but a small number of ICs and transfer cells (TCs); Almost 58% OCCs and 42% ICs but no TCs in the third order veins; Nearly 87% ICs, 13% OCCs and still no TCs in fifth order veins (minor veins, Fig. 2a, b, Ma et al. 2019). 4 GolSs were isolated in cucumber, and *CsGolS1* was expressed highly in mature leaves, especially in companion cells of third and fifth order veins, suggesting its role in phloem loading preliminarily. The stachyose export was decreased but sucrose was increased in petiole of *CsGolS1*-RNAi plants, and the trend was opposite in *CsSUT2*-RNAi plants, indicating the cucumber phloem loading obeys not only the polymer trapping model but also the apoplastic loading strategies (Ma et al. 2019). Comparably, interference of the cucumber *CsSTS*, which localized in CCs, altered stachyose content and upregulated *CsSUTs* expression (Lü et al. 2017). As a matter of fact, the mixed phloem loading pathway was present in many plants, such as in *Scrophulariaceae*, based on the co-existence of ICs and OCCs in the minor veins (Voitsekhovskaja et al. 2006; Turgeon and Medville 2011). In *Amborella trichopoda*, *AmSTS* and *AmSUT1* were expressed in ICs and CCs of the same veins, respectively (Knop et al. 2004; Voitsekhovskaja et al. 2009). In an RFOs-transporting species, *Fraxinus excelsior*, the *FeSUT1* was also found in SEs and the OCCs of minor veins (Öner-Sieben and Lohaus 2014; Öner-Sieben et al. 2015). How the combination of two phloem loading strategies can benefit the phloem loading process and plant growth of these species remains to be further studied.

Up to now, we still do not know the evolutional mechanism of RFOs translocating plants, and we cannot see the back diffusion of raffinose and stachyose in polymer trap plants, there must exist the reasons why RFOs were segregated. The most possible explanation is that the growth potential was increased by the high export sugar rate, and herbivory was limited by the minimizing total carbohydrate concentration in the mesophyll (Turgeon 2010; Cao et al. 2013).

### RFOs phloem unloading in sink organs

After phloem loading followed by the long-distance transport, the phloem sap would be unloaded into sink organs. Phloem unloading is a process that the nutrients (sugar, amino acid, ions), water, and signaling molecules (RNAs, proteins, phytohormones) move from SE/CCs complex to sink parenchymal tissue by the cell-to-cell delivery. The phloem unloading mechanisms mainly including two pathways, symplasmic transport that depends on the intercellular plasmodesmata and apoplasmic transport which needs to go across the intercellular plasma membrane through sugar transporters. Also, the phloem unloading pathway maybe shifted from one to the other and even switched for several times based on the fruit type, fruit structure, and fruit developmental stages (Nie et al. 2010; Braun et al. 2014; Zhang et al. 2017). On the one hand, the continuous phloem unloading of sugar can satisfy the nutrients demand of sink growth; on the other hand, the transported sugar acts as a signal molecule to regulate plant development (Ruan 2014). In this section, we mostly focused on the carbohydrate unloading, especially RFOs unloading pathway.

The symplasmic phloem unloading is a passive process that sugars flow from the SE/CC complex to the parenchyma cell (PPCs) following the concentration gradient. The sucrose and hexoses cleaved by invertase and sucrose synthase (SUS) would move through plasmodesmata to supply nutrients or stored in sink cells after synthesis of insoluble macromolecules (Ma et al. 2018). In the symplasmic manner, the aperture size of the plasmodesmata pores, which can be quantitatively described as the size exclusion limit, strongly regulates the movement of freely diffusing sugars (De Storme and Geelen 2014). Convincing evidence demonstrates that the deposition of callose at the plasmodesmata neck region constricted plasmodesmata aperture of the trans-plasmodesmata cytosolic channel, thereby limiting symplasmic permeability between neighboring cells (Chen and Kim 2009; Zavaliev et al. 2011). During the early cotton fiber elongation stage, the symplasmic pathway is predominant and the apoplasmic unloading manner for sucrose transport is switched on during later development to compensate as the deposition of callose in plasmodesmata prevented the symplasmic pathway (Zhang et al. 2017).

Compared to symplasmic phloem unloading, apoplasmic pathway is an energy-dependent, transporters-mediated, and active process that the sugars against the concentration gradient from SE/CC complex to PPCs. Many plants utilize apoplasmic phloem unloading pathway over the course of fruit development, such as kiwifruit (Chen et al. 2017), apple (Zhang et al. 2004), and pear (Zhang et al. 2014a, b). After entered apoplast by SWEETs, the sucrose was either directedly unloaded by SUTs into adjacent PPCs or cleaved by cell wall invertase and transported by hexose transporters (HTs) into adjacent PPCs.

**Fig. 3:**
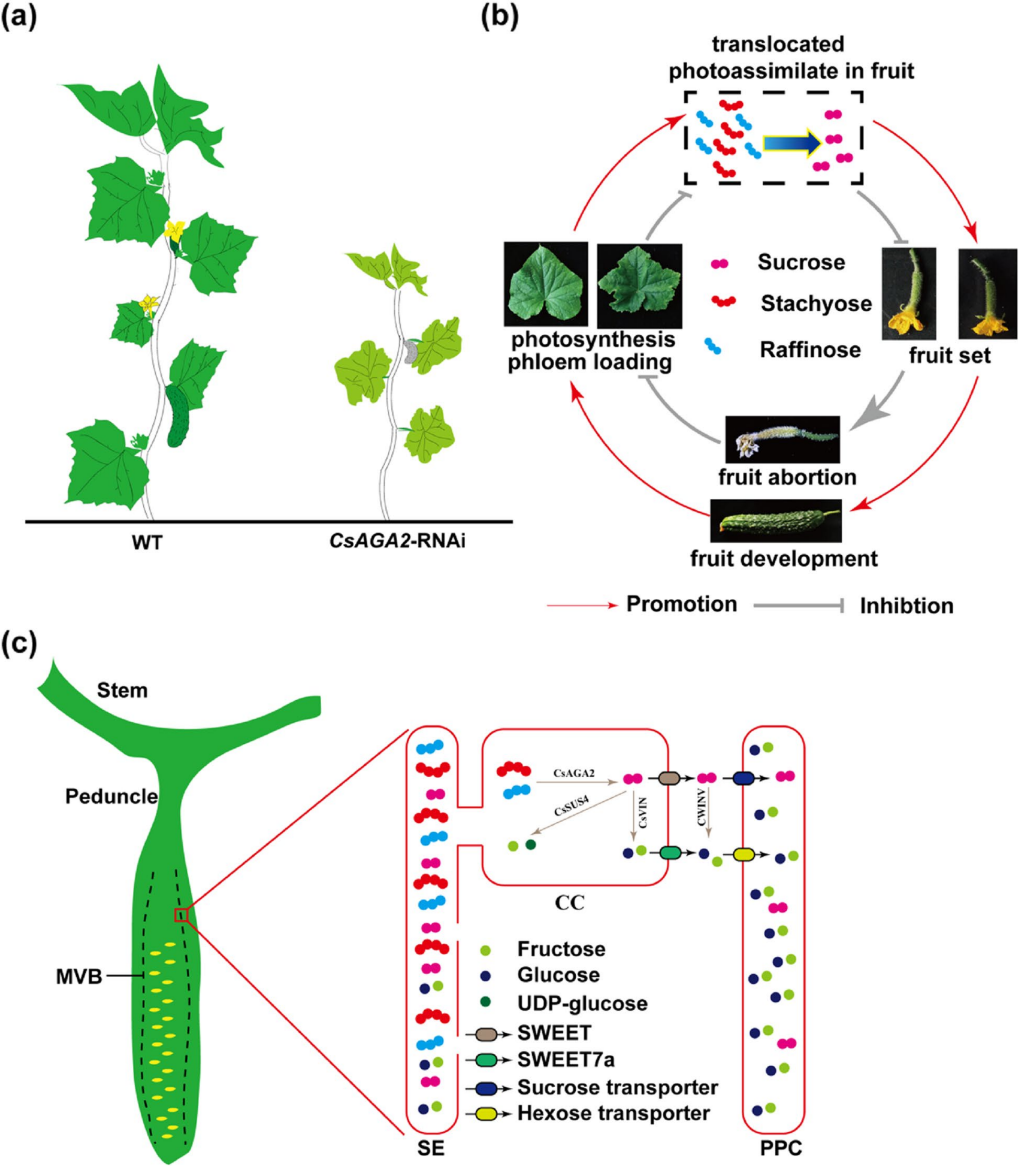
The phloem unloading pathway of cucumber. **a** The schematic diagram of cucumber plants when altered *CsAGA2* expression. **b** The feedback regulation of sink organs on source leaves around *CsAGA2*. **c** Model cucumber fruit phloem unloading. Sugar (mostly stachyose and raffinose with a small amount of sucrose) synthesized in source leaves is transported to and unloaded in sink organs, as fruit. Some of the stachyose and raffinose are hydrolyzed to sucrose by α-galactosidase (α-Gal) in the release phloem from the peduncle to gynophore and fruit MVB, and then may be broken down into fructose and UDP-glucose by SUS or into fructose and glucose by INV in companion cells. CsSWEET7a can further export hexoses to the apoplasmic space. Cell wall acid invertase (CWINV) would most likely break down sucrose into fructose and glucose in the apoplasm. The apoplasmic hexoses could be transported into phloem parenchyma cells (PPCs) by hexose transporters (HTs). There also may exist sucrose unloading pathway mediated by other sucrose exporters and importers. Abbreviation: MVB, main vascular bundle; SE, sieve element; CC, companion cell; PPC, phloem parenchyma cell

As mentioned above, the *Cucurbitaceae* plants are RFOs translocating species and there were mostly none RFOs in cucumber fruit (Hu et al. 2009), watermelon apical region (Hu et al. 2016), and melon fruits (Lingle and Dunlap 1987; Mitchell et al. 1992), indicating the RFOs hydrolysis occurred in sink organs. Stachyose and raffinose are rapidly hydrolyzed into sucrose and galactose after arriving at the melon fruit by α-galactosidases (Gao and Schaffer 1999). Using isotopic tracing method, the α-galactosidases were found to hydrolyze RFOs beginning at peduncle in cucumber (Ohkawa et al. 2010), but we also found the expression of an alkaline α-galactosidase2 (*CsAGA2*) in CCs of main vascular bundle (MVB), placenta and funiculus, down-regulation of *CsAGA2* induced fruit abortion and feedback regulation on source leaves, indicating the important role of *CsAGA2* on sink-source communication (Hua et al. 2021; Liu et al. 2022) (Fig. 3a, b). Briefly, when the CsAGA2 function was altered, the fruit would be aborted, which caused reduced phloem loading efficiency; While when the CsAGA2 works normally, the fruit would set, which in turn can lead to improved photosynthesis and phloem loading efficiency (Fig. 3a, b). Similarly, the watermelon fruit vascular bundle expressed but not in carpopodium *ClAGA2* also could influence fruit development and seed germination (Ren et al. 2021). Among all the melon α-galactosidases, the *CsAGA2* homologous gene *CmAGA2* presents much higher expression during fruit ripening (Dai et al. 2011).

After the RFOs hydrolysis in CCs, the sucrose was ready to be unloaded. For the SE/CC complex and PPCs are symplastically isolated, sugars were transported into apoplastic space following apoplasmic pathway (Hu et al. 2011; Ren et al. 2020). In watermelon, the vacuolar sugar transporter 1 (*ClVST1*) was shifted from the wild species vacuolar membrane to plasma membrane of the cultivar, and it unloaded both sucrose and glucose into apoplastic space in fruit VB. After then, the *ClSWEET3* takes up hexoses into fruit parenchymal cells followed by cell wall invertase (CWIN) hydrolyzed sucrose into glucose and fructose. Interestingly, the hexoses were synthesized again into sucrose and would be transported into vacuolar to store by tonoplast sugar transporter 2 (*ClTST2*) (Ren et al. 2018; 2020; 2021). In melon, the *CmTST2* also contributed to sucrose accumulation in vacuolar (Cheng et al. 2018). To date, any sucrose transporters in CCs of cucumber fruit vascular bundle were not identified functioning in phloem unloading, but a CCs localized sucrose synthase (*CsSUS4*) role in fruit development, and cytosol invertase (*CsCINV1*) in MVB was response to the down- or up-regulation of *CsSWEET7a* which is a hexose transporter in CCs of fruit MVB and induces the fruit expansion through export hexose into apoplastic space (Fan et al. 2019; Li et al. 2021) (Fig. 3c).

All these results demonstrate that the RFOs metabolism obeys the principle that “transport, unloading, and storage” were proceed simultaneously in most of the *Cucurbitaceae* fruit. Concerning that why cucumber fruit is less sweet than that of melon and watermelon, one reason is the time of eating, cucumbers were harvested in the commodity mature stage about 9 days after anthesis (DAA) while melon and watermelon were in the physiologically mature stage about 25 ~ 35 days after pollination (DAP); Another reason we guess is the TSTs transport sucrose into vacuolar and fruit mainly store sucrose in melon and watermelon (Wen et al. 2022), but cucumbers store predominately hexoses in mesocarp, the detailed evidence warrants further research.

To meet human demands, modern cultivars were evolute through involvement of the effective sugar distribution from source to sink to improve crop yield and quality (Julius et al. 2017). Simultaneous changes in sugar content, seed size, and oil content come in true by domesticating SWEET family sugar transporters in soybean (Wang et al. 2020). In cucumber and watermelon, many genes, such as *CsAGA2*, *CsSWEET7a*, *ClAGA2*, *ClSWEET3*, *ClVST1* and *ClTST2*, were located in the selective sweep regions during the speciation process between the wild ancestor and the semi-wild or cultivar group. The ancestors of cucumber and watermelon often yield small, bitter fruits with the lowest RFOs hydrolysis efficiency. Interestingly, the raffinose content in nonsweet wild watermelons was much higher than that of sweet watermelons. These results support that these genes were under selection during evolution and domestication, which led to the increased hydrolysis of RFOs, as well as subsequent sugar transport within the fruit.

Considering the fruit yield, quality, and genetic evolution, we have to further think about why RFOs are the main transport form other than sucrose in *Cucurbitaceae*. Most likely, the rapid increase of fruit weight could explain this question. The cucumber can grow from about 2.5 g to approximately 250 g in 8 ~ 10 days, and the watermelon from 2 g to about 5 kg within 20 days (Ma et al. 2018; Ren et al. 2021). In cucumber fruit, rapid cell division was initiated before anthesis and maintained to 5 DAA, while cell expansion was typically in action from 5 to 14 DAA, which stage was also the quick sugar accumulation period (Hu et al. 2009; Li et al. 2021). The sucrose content in watermelon fruit increased sharply from 18 to 26 DAP and accumulated about half of the total sugar in this short period. What's more exaggerated, the melon fruit could accumulate approximately total sucrose content within 10 days from 30 to 40DAP (Dai et al. 2011). Therefore, it is much more efficiency to increase the quality and yield of fruit crops through RFOs transport from phloem and hydrolysis.

### RFOs alter plants' response to abiotic stress

Apart from carbon storage and transportation in plants, RFOs also function in stress resistance. Continuous abiotic stress conditions, such as drought, salinity, and extreme temperatures, are major environmental disadvantageous factors that negatively influence the physiology and biochemistry of plants and limit crop production worldwide. To minimize the destructive effects caused by different types of abiotic stresses, plants have to evolve a series of mechanisms to sense, transduce signal (activation of signaling pathways), adapt (effect of gene expression levels), and defense, including the accumulation of amines, amino acids, or soluble sugars, like trehalose and RFOs in the cytosol or vacuole (Mahajan and Tuteja 2005; ElSayed et al. 2014).

RFOs can participate in osmotic adjustments as well as in membrane and protein stabilization. To maintain cell turgor and stabilize cell proteins under drought stress, RFOs was accumulated and functioned as osmolytes (Bartels and Sunkar 2005). During desiccation, the increased RFOs help to stabilize the membrane phospholipids in some resurrection plant species (Farrant et al. 2007). In tomato, RFOs can protect cellular integrity, *LeGolS-1* was accumulated during the seed desiccation stage to extend longevity and accumulated during seed germination to provide substrates for energy generation (Downie et al. 2003). Upregulated expression of *BnGolS* and *BnRS* genes in *Brassica napus* Heat Shock Factor (*BnHSF4a*) overexpression plants induced accumulation of raffinose and enhanced seed desiccation tolerance (Lang et al. 2017). In maize, *ZmHSFA2* and HEAT SHOCK BINDING PROTEIN 2 (*ZmHSBP2*) physically interact with each other and antagonistically regulated expression of *ZmGOLS2* and *ZmRS5*, overexpression of *ZmHSF2A* and *ZmHSBP2* enhanced and weaken plant heat stress tolerance by up- or down-regulation of raffinose synthesis, respectively (Gu et al. 2019). Frost tolerance in excised leaves of the common bugle (*Ajuga reptans L.*) correlates positively with the concentrations of RFOs (Peters and Keller 2009). In cucumber, *CsGolS*, *CsRS* and *CsSTS* were responded to chilling by increased expression and enzyme activity, and recovery to normal after removal of cold (Sui et al. 2012; Lü et al. 2017; Gu et al. 2018). And especially *CsGolS1* improves cucumber performance under cold stress by enhancing assimilate translocation (Dai et al. 2022), while CsGolS4 response to drought and cold simultaneously (Ma et al. 2021). Otherwise, down-regulating α-Galactosidase enhances freezing tolerance in transgenic Petunia by accumulation of RFOs (Pennycooke et al. 2003). *CsAGA2* and *CsAGA3* were induced again when the temperature recovered to normal after decreased expression in chilling (Gu et al. 2018). All these results concluded that the RFOs role in stress acting as osmoprotectants.

It has also been hypothesized that galactinol and RFOs can act as signals in mediating stress responses. Substantial evidence proved that galactinol and also raffinose act as signals during pathogen-induced systemic resistance, supporting their role in defence against biotic stresses by activation of plant defence genes transcripts directly or through salicylic acid (Kim et al. 2008; Chaouch and Noctor 2010). Both galactinol and raffinose were proposed to function as endogenous signals downstream of ROS and trigger ROS responses to cellular functions and lead plants to acclimation or cell death (Valluru and Van den Ende 2011). As mentioned above, unfavorable conditions like cold temperatures or drought significantly increase raffinose synthesis genes expression and synthesize RFOs. Although it is rather disadvantageous for sucrose distribution from photosynthetic source tissue to the phloem of sucrose-translocating plants, production of stress-induced RFOs in sinks are quite fine-tuned mechanisms to allow proper functionality of cellular and long-distance transport processes, and positively affect plant yield (Keller et al. 2021). But in RFOs-translocating plants, like cucumber, the RFOs occupied 57.11% of the translocated sugars in phloem sap, down-regulation of *CsAGA2* caused RFOs accumulation in peduncle and MVB, leading to reduced photosynthesis and abnormal leaves (Liu et al. 2022). We proposed that if the sink strength is limited, RFOs could be as signaling to inform source leaves about the current phloem flow status in return to activate an efficient feedback mechanism. Whether RFOs function as signaling directedly and how the signaling was achieved warrants further research.

Identifying and understanding the physiological and molecular mechanisms of plant adaptation to abiotic stress is important for agronomy and economy. In this section, we recapitulate the function of RFOs with its synthase and hydrolysis enzymes in osmotic adjustment and signaling pathways in response to stress. In addition, RFOs could also participate in the tolerance of reactive oxygen species (ROS) as antioxidants (Nishizawa et al. 2008; Salvi et al. 2018). However, it is demonstrated that changes in RFOs content in response to stress are part of a mechanism for carbon storage rather than protection in certain plant species (ElSayed et al. 2014), further study needs to be done to better assess RFOs functional relevance in response to abiotic stress.

## Conclusions and perspectives

RFOs function in plants diversely as they are used as storage compounds in seeds, as long-distance transport sugars of *Cucurbitaceae*, *Lamiaceae*, *Oleaceae* and *Scrophulariaceae* families, and as osmolytes or signaling for protection against a series of abiotic stresses. Phloem loading acts as a limiting step for the sugar utilization at the source organs, and the strategy of sucrose transport in leaves has long been discussed. In this review, we shed light on the mechanism of RFOs biosynthesis and phloem loading which is dependent on the polymer trapping model. Meanwhile, the RFOs unloading as well as its role in stress resistance were summarized.

Recently, research about TCs suggested its cell wall ingrowth is much more rely on the phloem loading activity in *Arabidopsis*, higher cytosolic sucrose concentrations from MCs transported through plasmodesmata promote such ingrowths, while exogenous sucrose increases apoplastic sucrose levels sufficiently results in lower wall ingrowth deposition (McCubbin and Braun 2020; Wei et al. 2020). In cucumber, the ICs and OCCs were the main cells to load sugars, TCs were found only in main veins that mainly function in sugar transport other than phloem loading, what is the role of TCs in cucumber worthy of exploration? In addition, it will go through transition from sink to source of leaves (Turgeon and Webb 1973; Savage et al. 2013), but what controls the shift and how the phloem cell anatomy changes still puzzled researchers. Evolution of the molecular physiology of RFOs biosynthesis and hydrolysis are still mysterious, and more insights into the evolution are needed to resolve.

Phloem sugar unloading partly determines the sink strength, sufficient nutrients supply could improve crop yield and quality. Different from sucrose and hexoses, no transporters were identified to transport RFOs, indicating the importance of α-galactosidase in cleavage of RFOs into sucrose and galactose. On the one hand, once the α-galactosidase was un-functioned, how the plants deal with the accumulated RFOs in sink organs is unknown. What’s more, little galactose was detected in cucumber fruit, where they functioned, we guess it was quickly into cell wall biosynthesis process to fulfil cell expansion and proliferation, but evidence needs further study. On the other hand, how the accumulated RFOs in sinks delivery signaling to source leaves to finish feedback regulation, if it is through themselves or through FLOWERING LOCUS T (FT) to activate or in activate other genes like FT-paralog *StSP6A* induces potato tuberization by inactivation of the stolon-localized *StSWEET11* to block sucrose efflux into the apoplasm and accelerates symplasmic sucrose unloading to promote tuber development (Navarro et al. 2011; Abelenda et al. 2019).

To protect plants from stress damage, they had evolved a set of strategies, including accumulation RFOs. We evaluated and described the literature available on GolS, RS, STS, and α-galactosidase gene expression as well as RFOs accumulation in response to stress. In fact, the stress influences resource allocation from source to sink tissues whether in sucrose- or RFOs-translocating plants, it is questioned how to distinguish whether RFOs accumulation is a storage or withstand to stress. Heterologous expression of melon *CmGolS*, cucumber *CsRS*, and *Alonsoa AmSTS* in *Arabidopsis* not only introduce RFOs in the main translocation stream but also decreased fecundity of fed aphid by the choice of them on sucrose other than RFOs directed toxicity (Cao et al. 2013). It provides us with the possibility that RFOs-translocating plants evolved from long time ago to avoid biotic damage and improve transport efficiency, but accurate evidence needs to study future. Furthermore, the existence of RFOs in sucrose-translocating plants reminded us to explore how they were metabolized during source and sink communication.

## Data Availability

The authors declare that all data supporting the findings of this study can be found within the paper. Additional data supporting the findings of this study are available from the corresponding author upon request.
